# The donation-transplantation process and corneal graft failure: A case-control study

**DOI:** 10.1371/journal.pone.0321225

**Published:** 2025-05-22

**Authors:** Giovanna Karinny Pereira Cruz, Marcos Antonio Ferreira Júnior, Viviane Euzébia Pereira Santos, Allyne Fortes Vitor, Oleci Pereira Frota, Elen Ferraz Teston, Felipe Machado Mota, Maria Eduarda Gonçalves Zulin, Gustavo Moura Maidana, Letícia Lima Meza, Bruna Dias Abes, Mayra Dias

**Affiliations:** 1 Federal University of Rio Grande do Norte, Natal, Rio Grande do Norte, Brazil; 2 Federal University of Mato Grosso do Sul, Campo Grande, Mato Grosso do Sul, Brazil; Singapore National Eye Centre, SINGAPORE

## Abstract

**Introduction:**

The survival after a corneal transplantation depends on several factors, many of which are not fully known.

**Objective:**

The present study aimed to verify the relationship between the factors inherent to the donation-transplantation process in graft failure.

**Materials and methods:**

Longitudinal case-control study carried out in a reference service in corneal transplantation in northeastern Brazil, which included transplanted patients with corneal graft failure and their respective controls. The sample consisted of 27 cases of graft failure and 54 controls.

**Results:**

A total of 427 transplantations were performed between 2010 and 2016 in the studied service, during which 27 cases of corneal graft failure were identified, representing a failure rate of 9.04%. The intergroup descriptive analysis identified the predominance of corneal graft failure in females (cases: 59.26%; control: 55.56%), brown (cases: 65.38%; controls: 53.70%), single (cases: 71.43%; controls: 68.52%) and residents in the capital and metropolitan region (cases: 66.67%; controls: 50%). The mean age of the patients who underwent corneal transplantation was 52.31 years (cases: 56.15 years; control: 50.39 years). Regarding the “type of keratoplasty”, the prevalence of penetrating keratoplasty was observed in 87.65% of the transplantations (cases: 81.48%; controls: 18.52%). In addition, 85.19% of the failures were late and 46.91% of the patients subjected to transplantation had already undergone an ophthalmological surgical procedure previously.

**Conclusion:**

The present study identified the following variables as factors of the donation-transplantation process associated with corneal graft failure: donor button size ≤ 8.25 mm, donor-recipient button size difference less than 0.25 mm, death due to respiratory failure, time interval between enucleation-preservation and corneal endothelial disorder.

## Introduction

The cornea is a layer of avascular and transparent connective tissue located anteriorly to the eyeball that assists in improving the image formed by the retina. When presenting structural dysfunctions that lead to the formation of scars, opacifications or irregularities in this tissue, there may be a high degree of impairment in vision and even blindness [1,2].

Among the procedures that can restore the affected vision, one can cite keratoplasty, which has different techniques depending on the degree of tissue involvement. When replacing the corneal tissue in its entirety, the surgical technique is called penetrating keratoplasty. If only the affected lamella is replaced, the procedure is called lamellar keratoplasty [3].

Keratoplasty can present high success rates, but it is not without risks, and there may be intraoperative problems, such as inadequate graft centralization, suprachoroidal hemorrhage, infection and damage to the surrounding ocular structures, as well as postoperative risks, such as transplantation wound dehiscence, infection, graft rejection, disease recurrence, severe astigmatism and corneal graft failure (CGF) [4].

CGF is characterized by loss of tissue transparency, which consequently leads to loss of visual capacity [5]. Among the main characteristics for the success of corneal transplantations, one can cite their immunological privilege; however, when there are failures in the natural protection mechanisms of this tissue, graft rejection occurs, which is still the most common cause of CGF[6].

Nonetheless, rejection is not the only possible cause for CGF, since this outcome can occur due to multifactorial conditions, not fully understood, which makes it necessary to search for evidence on these predictive factors.

Knowledge of the epidemiological and clinical profile of corneal transplantations allows us to identify risk groups for the purpose of prevention and implementation of care that result in better prognoses [7].

The present study aims to verify the relationship between the factors inherent to the donation-transplantation process in graft failure.

## Materials and methods

This is a study with a quantitative, analytical, longitudinal, **hospital-based** and **case-control** approach, which was carried out at the Onofre Lopes University Hospital, belonging to the Federal University of Rio Grande do Norte (UFRN), a reference service in Corneal Transplantation in the state of Rio Grande do Norte (RN), located in the northeastern region of Brazil.

To structure the report of this study, the *Strengthening the Reporting of Observational Studies in Epidemiology* (STROBE) initiative was used, developed for the construction and evaluation of epidemiological studies [8].

In this study, the selection of comparison groups in a paired manner was used as a classification criterion, where the adopted outcome variable was “corneal graft failure”.

The definition of the pairing variables for the composition of the control group in relation to the case group occurred through the identification of confounding variables, based on a review study that was previously carried out with the objective of identifying predictors of corneal graft failure. Thus, the following were chosen as potential confounding variables for the present study: corneal vascularization, glaucoma and rejection.

For the purposes of this study, two controls were considered for each case (2:1). Thus, the final sample consisted of a case group (n = 27) and a control group (n = 54).

The **case group** was composed of patients who underwent CT with a postoperative diagnosis of corneal graft failure, while the **control group** was composed of patients who underwent transplantation without a diagnosis of corneal graft failure.

The case-control study population consisted of the medical records of patients who underwent CT followed by the HUOL Ophthalmology service. The sample was formed through the non-probabilistic sampling process, consisting of the medical records of patients who underwent corneal transplantation in the service from January 2010 to the end of 2016 and were subsequently monitored for continuity of care in the same service. Data from patients of all ages, of both sexes, regardless of the clinical condition indicating registration in the National Transplantation System (NTS), were included.

Regarding sociodemographic data, the following variables were addressed: gender, race, marital status, place of residence and age of the recipient, in addition to sex and age of the donor.

The considered clinical and surgical variables intrinsic to the donation-transplantation process were: corneal quality, type of keratoplasty, type of graft failure, recipient ocular diagnosis, type of corneal disorder, operated eye, surgical purpose, type of surgery, lens condition, surgery combined with cataract extraction, previous ocular surgery, intraoperative complications in transplantation, donor-recipient sex difference, captured eye, donor corneal button size, recipient corneal button size, donor-recipient corneal button size difference, preservation media, physiology of death, cause of death, tissue purpose, time interval between death and eyeball nucleation, time interval between death and corneal preservation, time interval between death and transplantation and time interval between corneal enucleation and preservation.

For the variable “corneal quality”, the classification given by the eye bank team was considered after evaluating the corneal tissue in a slit lamp at two moments (two biomicroscopic evaluations by ophthalmologists). Accordingly, the tissues were classified as “excellent”, “good”, “regular” or “bad” for keratoplasty. The first evaluation moment occurred after the enucleation and immersion of the corneal tissue in a preservation media. The second moment corresponded to the final evaluation of the tissue to release the cornea for transplantation or disposal. The evaluation of the corneal tissue can only be performed by professionals trained by the Pan-American Association of Eye Banks (PAAEB); therefore, all corneas in this study were evaluated by professionals qualified for this purpose. The classification of the corneal quality attributed by the ophthalmologists of the Eye Bank under study after the evaluations took into account 13 criteria, namely: senile arch, scars, epithelial defect, epithelial exposure, stromal infiltrate, subepithelial opacity, pterygium, Descemet’s membrane folds, stromal edema, stromal streak, guttata, specular reflex and endothelial cell loss. The applied ophthalmological criteria were chosen by the PAAEB and by the Eye Bank Association of America (EBAA) as requirements that make up the evaluation of corneal tissue in the HMETBs.

For descriptive analysis and application of statistical tests, the Statistical Package for the Social Sciences (SPSS) software, version 25.0, was used for testing. In the comparison of the case and control groups with sociodemographic and clinical characteristics of the patients, the Chi-Square and Fisher’s Exact tests were applied for categorical variables, while the Mann-Whitney test for quantitative variables. Measures of effect magnitude by calculating the Odds Ratio (OR) were analyzed for variables that showed a statistically significant relationship. After the bivariate analysis, a final adjustment model was built by logistic regression, using the Backward Wald Stepwise method. The significance level for all analyses was 0.05.

Data collection was performed after approval of the research protocol by the Research Ethics Committee of the Federal University of Rio Grande do Norte, under the Certificate of Presentation of Ethical Appreciation number 80007117.8.0000.5537 and the favorable Opinion number 2.454.077. The data was accessed for research purposes from 02/01/2018. All data were made completely anonymous before access.

## Results and discussion

The presented results expose the epidemiological and clinical profile of patients with corneal graft failure and verify the relationship between the factors of the donation-transplantation process in graft failure, in order to answer the research question: which factors of the donation-transplantation process are related to corneal graft failure?

### Clinical and epidemiological profile of patients who underwent keratoplasty and corneal graft failure

A total of 427 transplantation procedures were performed between 2010 and 2016 in the studied service, during which 27 cases of corneal graft failure were identified, approximately 3.86 cases of failure per year, with a failure rate of 9.04%.

The intergroup descriptive analysis (27 cases and 54 controls) identified the predominance of corneal graft failure in females (cases: 59.26%; control: 55.56%), brown (cases: 65.38%; controls: 53.70%), single (cases: 71.43%; controls: 68.52%) and residents in the capital and metropolitan region (cases: 66.67%; controls: 50%).

The mean age of the patients who underwent CT was 52.31 years (cases: 56.15 years; control: 50.39 years). The minimum and maximum ages were 9 and 88 years, respectively.

In the inferential analysis of comparison between the case and control groups, the variables “gender”, “age group”, “place of residence”, “race” and “marital status” did not show evidence of a statistically significant relationship between the groups.

The clinical profile of the patients highlights the variables “type of keratoplasty”, where the prevalence of penetrating keratoplasty surgeries was observed in 87.65% of the transplantations (cases: 81.48%; controls: 18.52%); “type of failure” with 85.19% of late failures and 14.81% with primary graft failure. “Previous surgery”, where 46.91% of the patients subjected to transplantation had previously undergone an ophthalmological surgical procedure (cases: 51.85%; controls: 44.44%). The ophthalmological surgical procedure of lens removal (facetectomy) was previously performed in 33.33% of the patients who developed graft failure.

Regarding the main ocular diagnoses that corresponded to the indicative conditions for transplantation, bullous keratoplasty was the main indication (30.86%), followed by keratoconus (20.99%). Regarding the distribution of disorders by the affected layer of the cornea, it was found that 53.09% were stromal disorders (cases: 37.04%; controls: 61.11%) and 46.91% endothelial (cases: 62.96%; controls: 38.89%). The surgical purpose of most corneal transplantations was optical (75.31%).

The variables used in the pairing (vascularization, glaucoma and rejection), although described, had no effect on the inferential analysis due to their control to avoid confounding bias. Thus, these variables were not used in the inferential analysis, since they are risk factors for graft failure as described in the literature.

To calculate the probability of statistical difference between the case *versus* control groups, the clinical variables “type of corneal disorder”, “surgical purpose”, “type of keratoplasty”, “type of surgery”, “lens”, “surgery combined with cataract extraction”, “previous surgery” and “intraoperative complication” were analyzed, as **shown in Table 1**.

**Table 1:** Clinical characteristics of the patients who underwent corneal transplantation from the case and control groups. Natal/RN, Brazil, 2023 (n = 81).

Clinical characteristic	Groups		p	Odds ratio [95% CI]
	Case (n = 27) n (%)	Control (n = 54) n (%)		
Type of disorder				
Stromal disorders	10 (37.04)	33 (61.11)	0.041^(1)^	**0.37** [0.14; 0.97]
Endothelial disorders	17 (62.96)	21 (38.89)		
Surgical purpose				
Optical	21 (77.78)	40 (74.07)	0.716^(1)^	**1.22** [0.41; 3.65]
Tectonic	06 (22.22)	14 (25.93)		
Type of keratoplasty				
Penetrating	22 (81.48)	49 (90.74)	0.288^(2)^	**0.45** [0.12; 1.71]
Lamellar	05 (18.52)	05 (9.26)		
Type of surgery				
Optional	22 (81.48)	38 (70.37)	0.282^(1)^	**1.85** [0.60; 5.75]
Emergency	05 (18.52)	16 (29.63)		
Crystalline				
Phakic	18 (66.67)	36 (66.67)	0.874^(1)^	---
Aphakic	01 (3.70)	01 (1.85)		
Pseudophakic	08 (29.63)	17 (31.48)		
Combined surgery				
Yes	02 (7.41)	02 (3.70)	0.597^(2)^	**2.08** [0.28; 15.63]
No	25 (92.59)	52 (96.30)		
Previous ophthalmological surgery				
Yes	14 (51.85)	24 (44.44)	0.529^(1)^	**1.35** [0.53; 3.40]
No	13 (48.15)	30 (55.56)		
Intraoperative complication				
Yes	03 (11.11)	06 (11.11)	1.000 ^(1)^	**1.00** [0.23; 4.35]
No	24 (88.89)	48 (88.89)		

Caption ^(1)^ Chi-Square Test. ^(2)^ Fischer’s Exact Test. CI: Confidence Interval.

There was a statistically significant difference between the case and control groups with the type of endothelial corneal disorder. Patients who had stromal disorders had a 37% lower chance of developing post-transplantation CGF compared to those who developed endothelial disorders. The other variables did not present a statistically significant relationship in relation to corneal graft failure.

Multiple logistic regression was used for multivariate analysis. The inclusion criterion for inserting the explanatory variables in the model was the identification of a value of p < 0.20 in the bivariate analysis. The investigation of the associated factors was done by means of classical multiple logistic regression, using the Stepwise method of the Backward Wald type.

The final model of multiple logistic regression, using the Wald test, for a significance level of 5% presented statistical evidence of association of the case and control groups with a type of corneal disorder. It was observed that the chance of patients with stromal disorders developing graft failure decreased by 65% compared to patients with endothelial disorders.

### The donation process and the relationship with corneal graft failure

In addition to characterizing the event of corneal graft failure by time, place and person, the present study addressed the possible causes for the event and why people develop graft failure regarding the factors inherent to the donation process.

In the total of 81 donors of the transplanted corneas in the analyzed sample, 79.01% were males (cases: 81.48%; controls: 77.78%), 45.68% of the corneal grafts came from left eyeballs (cases: 59.26%; controls: 51.85%), with a mean corneal button size of 8.56 mm (cases: 8.31; control: 8.68), while the recipient corneal button had a mean size of 8.19 mm (cases: 7.98; controls: 8.28). The mean difference in size between the donor-recipient was 0.42 millimeters (cases: 0.35; controls: 0.46). The mean age of corneal donors was 43.85 years (cases: 44.93; controls: 46), half of the donors were up to 46 years (cases: 46 years; controls: 47 years).

Optisol was the preservation media used in 95.06% (cases: 92.59%; controls: 96.30%) of the tissues collected for transplantations. As for quality, 1.23% of the corneas were classified as excellent, 71.61% as good, 20.99% as regular and 6.17% as poor. For inferential analysis, the variable “corneal quality” was regrouped as follows: “excellent/good” (cases: 59.26%; controls: 79.63%) and “regular/poor” (cases: 40.74%; controls: 20.37%). Most corneas were indicated for optical transplantations (91.36%) (cases: 96.30%; controls: 88.89%). The main cause of death of donors was through a pathophysiological mechanism of cardiorespiratory arrest (66.67%) (cases: 74.07%; controls: 62.96%).

To calculate the statistical difference between the case and control groups with the factors related to the donation process, the following categorical variables were used: donor sex, difference between donor-recipient sex, donor age, difference in the size of the donor-recipient corneal button, physiology of death, cause of death, preservation media, tissue purpose and corneal quality, as shown in **Table 2**. Additional information is available in the supporting files S1 Table and S2 Table.

**Table 2:** General characteristics of the donation process of the corneal transplantation recipients from the case and control groups. Natal/RN, Brazil, 2023 (n = 81).

Characteristics	Group		p	Odds ratio [95% CI]
	Case (n = 27) n (%)	Control (n = 54) n (%)		
Donor gender				
Male	22 (81.48)	42 (77.78)	0.700^(1)^	**26** [0.39; 4.03]
Female	05 (18.52)	12 (22.22)		
Difference between donor and recipient sexes				
Yes	15 (55.56)	24 (44.44)	0.345^(1)^	**1.56** [0.62; 3.96]
No	12 (44.44)	30 (55.56)		
Donor age				
Up to 45 years	13 (48.15)	26 (48.15)	1.000^(1)^	**1.00** [0.39; 2.52]
above 45	14 (51.85)	28 (51.85)		
Difference in button size between donor and recipient^*^				
Less than 0.25	12 (52.17)	07 (13.46)	0.001^(1)^	---
Equal to 0.25	11 (47.83)	43 (82.69)		
Above 0.25	---	02 (3.85)		
Physiology of death^**^				
CRA	20 (74.07)	34 (62.96)	0.317^(1)^	**1.68** [0.60; 4.67]
Cephalic death	07 (25.93)	20 (37.04)		
Cause of death*				
AMI/Shock	09 (33.33)	20 (37.04)	0.028^(1)^	---
	Brain stroke	08 (29.63)	15 (27.78)	
	Respiratory failure	04 (14.81)	---	
	TBI and other traumas	06 (22.23)	19 (35.18)	
Preservation media				
Optisol	25 (92.59)	52 (96.30)	0.597^(2)^	**0.48** [0.06; 3.61]
Eusol	02 (7.41)	02 (3.70)		
Cornea purpose				
Optical	26 (96.30)	48 (88.89)	0.415^(2)^	**3.25** [0.37; 8.47]
Tectonic	01 (3.70)	06 (11.11)		
Corneal quality				
Excellent/Good	16 (59.26)	43 (79.63)	0.052^(1)^	**0.37** [0.13; 1.03]
Regular/Poor	11 (40.74)	11 (20.37)		

Caption: ^(1)^ Chi-Square Test. ^(2)^ Fischer’s Exact Test. CI: Confidence interval; CRA: Cardiorespiratory arrest; AMI: Acute Myocardial Infarction; Brain Stroke: Brain Stroke; TBI: Traumatic brain injury. ^*^Some incomplete data. ^**^Multiple responses.

Evidence of a statistically significant difference between the case and control groups was found with the variables “cause of death” and “button size difference between donor and recipient”.

Through bivariate analysis, it was possible to identify that the cause of death due to respiratory failure and the difference in the size of the donor-recipient corneal button smaller than 0.25 mm were considered predictive factors for CGF.

**Table 3** below presents the analysis of the chronological variables related to the donation process, which were later analyzed to compare the difference between the patients from the case and control groups.

**Table 3:** Descriptive profile of the chronological variables of the donation process. Natal/RN, Brazil, 2023 (n = 81).

Variable	Minimum	Maximum	Median	Mean	SD	CV	P
Time between death-enucleation (in minutes)	30.00	1.385.00	235.00	291.17	251.31	86.31	<0.001
Time between enucleation-transplantation (in days)	3.00	14.00	10.00	9.54	2.73	28.56	<0.001
Time between enucleation-preservation (in minutes)	10.00	425.00	55.00	88.36	82.90	93.83	<0.001
Time between death-preservation (in minutes)	45.00	1.380.00	320.00	361.75	229.82	63.53	<0.001
Time between preservation-transplantation (in days)	3.00	14.00	10.00	9.54	2.73	28.56	<0.001
Time between death-transplantation (in days)	3.00	14.00	10.00	9.85	2.74	27.85	<0.001

Caption SD: Standard Deviation. CV: Coefficient of Variation. Kolmogorov-Smirnov test to check data normality.

As for the chronological variables of the donation process, the following means were obtained between the time intervals: death-enucleation: 4 hours and 51 minutes (cases: 4h10min; controls: 5h10min), death-preservation: 6 hours (cases: 5h10min; controls: 6h27min), enucleation-preservation: 1 hour and 30 minutes (cases: 1h52min; controls: 1h16min), death-transplantation: 9.85 days (cases: 9.96 days; controls: 9.80 days), enucleation-transplantation: 9.54 days (cases: 9.74 days; controls: 9.44 days) and preservation-transplantation: 9.54 days (cases: 9.74 days; controls: 9.44 days). The mean time since tissue immersion in preservation media and transplantation was 9.54 days (cases: 9.74; controls: 9.44) (Table 4).

**Table 4:** Chronological and anthropometric characteristics of the donation process of the subjects from the case and control groups. Natal/RN, 2023 (n = 81).

Variable	Group	Minimum	Maximum	Median	Average	DP	CV	p
Recipient age (in days)	Case	24.00	80.00	56.00	56.15	18.17	32.36	0.266
	Control	9.00	88.00	52.00	50.39	22.59	44.83	
Donor age (years)	Case	13.00	65.00	46.00	44.93	15.60	34.71	0.558
	Control	2.00	64.00	47.50	43.31	16.25	37.53	
Time between death and enucleation (in minutes)	Case	30.00	1.385.00	205.00	251.22	245.88	97.87	0.118
	Control	45.00	1.345.00	250.00	311.15	253.89	81.60	
Time between enucleation-transplantation (in days)	Case	3.00	14.00	11.00	9.74	2.97	30.48	0.491
	Control	4.00	14.00	10.00	9.44	2.62	27.72	
Time between enucleation-preservation (in minutes)	Case	20.00	425.00	75.00	112.41	109.82	97.70	0.144
	Control	10.00	320.00	51.00	76.33	63.37	83.01	
Time between death- preservation (in minutes)	Case	45.00	770.00	265.00	310.30	160.75	51.81	0.168
	Control	75.00	1.380.00	347.00	387.48	254.99	65.81	
Time between preservation- transplantation (in days)	Case	3.00	14.00	11.00	9.74	2.97	30.48	0.491
	Control	4.00	14.00	10.00	9.44	2.62	27.72	
Time between death- transplantation (in days)	Case	3.00	14.00	11.00	9.96	3.03	30.43	0.657
	Control	4.00	14.00	10.00	9.80	2.62	26.71	
Donor button size (in millimeters)	Case	8.00	8.50	8.25	8.31	0.17	2.03	<0.001
	Control	8.00	13.00	8.50	8.68	0.86	9.96	
Recipient button size (in millimeters)	Case	7.50	8.50	8.00	7.98	0.17	2.09	0.064
	Control	7.50	13.00	8.00	8.28	1.04	12.51	

*Caption* SD: Standard Deviation CV: Coefficient of Variation. Mann-Whitney test.

There was evidence of a statistically significant difference between the anthropometric variable “donor button size” and graft failure. Patients with corneal graft failure had smaller donor button size (ROC curve: ≤ 8.25 mm, p < 0.001). The chronological variables related to the donation process did not present a statistically significant difference between the case and control groups in the bivariate analysis.

After bivariate analysis, multiple logistic regression was used for multivariate analysis. The inclusion criterion for inserting the explanatory variables in the model was the identification of a vapor of p < 0.20 in the bivariate analysis. Thus, the following variables were selected to choose the model: “cause of death”, “corneal quality”, “time between enucleation and preservation”, “time between death and preservation”, “time between death and enucleation”, “size of the donor corneal button” and “size difference of the donor-recipient button”. Additional information is available in the supporting file S3 Table.

To investigate the predictive factors for corneal graft failure, classical multiple logistic regression was used, based on one of the most applied methods in logistic regression, the Stepwise method of the Backward Wald type.

The final model of multiple logistic regression using the Wald test for a significance level of 5% identified the chronological variable “time between enucleation-preservation” as predictive factor for corneal graft failure. As for the chronological variables, it was obtained that, for every minute elapsed between enucleation and preservation, the chance of a patient developing graft failure increases by 1%.

The description of the epidemiological profile of cases of corneal graft failure in the present study showed a predominance of females, browns, singles, residents in the state capital or metropolitan region and mean age 56.15 years. The improvement in visual acuity corresponded to the clinical objective prevalent in keratoplasty surgeries (cases: 77.78%; controls: 74.07%).

The clinical profile of the patients who developed corneal graft failure (n = 27) identified a predominance of cases of late failure (85.19%). Late failure consists of the loss or absence of tissue transparency and consequent reduction of visual acuity in the mediate or late postoperative period, months or years after corneal transplantation [9,10].

The most common type of corneal disorder in cases of graft failure was endothelial in nature (cases: 62.96%; controls: 38.89%). Endothelial disorders have a higher risk of graft failure due to the regenerative incapacity of the endothelium and its main function, which is to maintain the cornea transparent. The main corneal diseases that present endothelial disorders are Fuchs’ dystrophy, posterior polymorphic dystrophy, hereditary congenital endothelial dystrophy, iridocorneal endothelial syndrome, bullous keratopathy and trauma [10–12].

Through bivariate analysis and logistic regression model, the “type of corneal disorder” was identified as a predictor variable for corneal graft failure. The chance of patients with stromal disorders developing corneal transplantation failure decreased between 37% (bivariate analysis) and 65% (logistic regression) in relation to patients with endothelial disorders.

The corneal endothelium does not have regenerative cellular properties, with an average cell loss of 0.6% per year. Therefore, the aging process or endothelial trauma are predictive factors for diseases that aggravate the corneal endothelium [7,12,13].

The predominance of individuals over the age of 50 in cases of graft failure can be justified due to the large number of diseases of the corneal endothelium being increasingly that become increasingly evident with increasing age [7,13].

As the aging process is constituted, there is a decrease in the number of corneal endothelium cells. This physiological event justifies the statistical association established in this study between endothelial corneal disorders and graft failure, since the main function of the corneal endothelium is to maintain graft transparency. Another factor in question is the recurrence of endothelial diseases of genetic etiology, which result in damage to the graft endothelium [10,14].

A study conducted in the USA identified that the longevity of the corneal graft is often determined by the survival of endothelial cells. The adoption of new surgical techniques improved graft survival. In cases where the host endothelium is healthy, retaining it with deep anterior lamellar keratoplasty can significantly reduce long-term endothelial cell loss. Endothelial keratoplasty and penetrating keratoplasty differ fundamentally in the relative rates of early and late loss of central endothelial cells. The rate of post-keratoplasty decline is influenced by the retention or replacement of the host endothelium by the donor endothelium, due to variations in surgical techniques and due to the characteristics of recipients and donors [15].

The relationship established between endothelial disorders and corneal graft failure should be taken into account in future research. Most studies choose to establish a relationship between corneal graft failure and ocular diagnoses or indicative conditions for corneal transplantation [16–20].

Clinical and epidemiological variables such as gender, age group (up to 54 years; over 54 years), place of residence, race, marital status, previous surgery, intraoperative complications in CT, pseudophakic/aphakic lens, surgery combined with cataract extraction and emergency transplantations did not show a statistically significant relationship with corneal graft failure. However, previous studies have pointed to some of these variables as predictors of graft failure, such as age group, previous surgeries, pseudophakic or aphakic eye and intraoperative complications [9,16–19].

In addition to the factors already discussed in the scientific literature as possible predictors for CGF, the demographic variable “race” deserves attention, although it did not present statistical significance. The higher incidence of brown individuals in the study sample may be associated with the characteristics of the population of the state where the research was carried out [21]. However, studies indicate that the Afro-descendant population of Brazil faces inequality in access to health services due to social, structural and cultural factors [22]. Accordingly, it is known that, among the possible causes that can lead to CGF, one can cite the lack of adherence of patients to post-transplantation follow-up consultations, which aim to be able to intervene in modifiable factors, such as glaucoma and vascularization of the recipient layer [23].

It is suggested that these factors should be investigated in future studies with larger samples, since one of the limitations of the present study is the sample universe, which may have compromised the identification of other risk factors for graft failure.

### The donation process and the relationship with corneal graft failure

The complication “graft failure” consists of the inadequate functioning of the corneal graft, with no response to the established function. The multifactoriality that involves corneal graft failure requires a thorough analysis of the determining factors for the occurrence of this event [9,24,25].

After analyzing the data through inferential statistics, the following factors in the corneal graft donation process were found to be associated with failure: donor button size ≤8.25 mm, donor-recipient button size difference less than 0.25 mm, death due to respiratory failure and time interval between enucleation-preservation.

Graft failure associated with donor corneal button size less than or equal to 8.25 mm should be accompanied by future studies to observe this occurrence in other samples, since corneal buttons from donors with a size of approximately 7.5mm are commonly found in clinical practice [10,26–28].

The obtained results corroborate studies carried out in Germany and South Korea that observed that the donor button with small or large dimensions can interfere with the final result of CT [10,26–28].

Data in the literature indicate that smaller grafts can cause astigmatism and larger diameters are associated with a greater tendency for the formation of peripheral anterior synechiae and increased intraocular pressure [10,26–28].

In addition, the mean size of the identified donor button was about 0.25 mm greater in diameter than that of the recipient. The proximity of the donor graft to the recipient limbus and increased exposure to the host immune system may be related to the high rate of graft failure in eyes with smaller corneas [10,26–28].

The main pathophysiological mechanism involved in the causes of death of donors was cardiorespiratory arrest (CRA); however, in the inferential analysis, CRA was not considered a predictive factor for failure. The association inferred by the bivariate analysis was with donors with deaths due to respiratory failure.

Oxygenation of corneal tissue occurs primarily through atmospheric oxygen. The peripheral portion of the cornea receives oxygen by diffusion from the limbal blood vessels, while the central portion uses atmospheric air. The cornea is exposed daily to hypoxia before the eyelid is opened and during the sleep cycle, where oxygen levels can drop from 21% to 8%. Even in the absence of blood and lymphatic vessels in healthy corneal tissue, the mechanism of tissue oxygenation is of fundamental importance for tissue hemostasis. Tissue hypoxia can compromise the mechanism of transparency and functioning of the cornea [29,30].

Respiratory failure may be associated with cases of GCF in which hypoxia sets in earlier compared to the other types of cause of death under analysis. (AMI, SHOCK, STROKE, TBI and other traumas). However, this association calls into question the importance of peripheral tissue oxygenation in the cornea, where the limbal blood vessels are located. Thus, tissue hypoxia is one of the factors associated with compromising the immune privilege of the cornea, as it causes an imbalance between angiogenic and anti-angiogenic factors that preserve corneal transparency [31].

This study found a statistically significant association between the chronological variable “time between enucleation-preservation” and corneal graft failure. It was observed that every minute between enucleation-preservation increases the chances in 1% of the individual who received this corneal tissue to develop graft failure. However, time intervals related to enucleation-preservation are not analyzed as criteria for capture or exclusion of viable corneal tissue for transplantation in Brazil.

Such relationship can be justified because the preservation media contains antiseptic and antibiotic properties that promote the maintenance of tissue functionality. The earlier the cornea comes into contact with the preservation media, the lower the likelihood of harmful events for the tissue [10,32–34].

The primary purpose of corneal preservation media is to maintain endothelial cell viability from the time donor tissue is obtained until transplantation [35].

Since tissue degradation begins after cessation of circulation, which in turn releases cytotoxic substances. It is assumed that, with increasing time after death, the quality of the donated tissues is affected. In addition, microbial flora continues to change qualitatively and quantitatively. Thus, it is recommended that corneal preservation occur as soon as possible after death [32–34].

Once recovered from cadaveric donors, human corneas must be preserved to maintain cell viability and tissue transparency, crucial parameters for later transplantation. Currently, there are two important preservation systems that are adopted worldwide: cold/hypothermic storage with preservation between 2 and 6° C, used mainly in America and Asia [36]. In the present study, the preservation system used by BTOH was hypothermic, whose Optisol was the preservation media chosen in 95.06% (cases: 92.59%; controls: 96.30%) of the tissues collected for transplantations.

Among the variables that did not behave as predictors of corneal graft failure during the inferential analysis, “corneal quality” deserves to be highlighted (p = 0.052). The variable “corneal quality” was excellent/good (cases: 59.26% versus controls: 79.63%) and fair/poor (cases: 40.74% versus controls: 20.37%) in the comparison between the groups. It is suggested that future studies, with a larger sample size, evaluate the behavior of this variable in relation to cases of corneal graft failure. Corneal quality is a prominent variable throughout the donation-transplantation process that deserves attention regarding its performance in relation to successful CT.

The donor age did not show a significant relationship with cases of failure (p = 0.558), which corroborates the study conducted in the USA entitled “Cornea Donor Study (CDS)”. The CDS aimed to evaluate the effect of donor age on graft survival and endothelial cell loss in penetrating keratoplasty for endothelial disease. At 5 years, there was no difference in graft survival (86%) between participants who received corneas from donors aged 12–65 and 66–75 years [18].

Based on the above regarding the donor age and its relationship with CGF, the hypothesis in question can be confirmed as valid: “the age of the advanced donor is a confounding factor for CGF and actually corresponds to a predictive factor for the decrease in the quality of the corneal tissue that will be donated” [37]. What actually causes CGF is endothelial dysfunction and not advanced donor age.

In vascularized organ transplantations, gender incompatibilities have higher rates of immune rejection, unlike the cornea, avascular tissue, whose donor-recipient gender incompatibility does not constitute unfeasibility for transplantation. Recent studies seek to investigate how gender mismatch behaves in CT [15,17,38]. This study did not identify a relationship between donor-recipient gender incompatibility and CGF

A publication of the UK Corneal Transplantation Registry pointed out that female patients with Fuchs’ dystrophy had better graft survival in cases where the obtained corneas were from female donors compared to those from male donors. Male recipients did equally well with both donors. On the other hand, a US cohort showed no relationship between gender incompatibility and CGF [17,38].

The survival after a keratoplasty depends on several factors, many of which are not fully understood. This multifactoriality represents a substantial risk for corneal tissue failure and consequently influence graft survival rates, especially if not identified and treated in a timely manner [39,40].

In view of the need to expand the production of longitudinal research, this study has contributed to the verification of the effects of predictors of post-transplantation graft failure, in such a way as to generate knowledge that supports the improvement of public policies and health care with regard to the early identification of risk factors for graft failure.

## Conclusions

The present study identified the following variables as factors of the donation-transplantation process associated with corneal graft failure: donor button size ≤ 8.25 mm, donor-recipient button size difference less than 0.25 mm, death due to respiratory failure, time interval between enucleation-preservation and corneal endothelial disorder

The success of corneal transplantation is subject to factors inherent to the corneal tissue donation and transplantation process. People with stromal disorders who have good quality corneal endothelium can guarantee preserved endothelial function by undergoing lamellar keratoplasty, given the importance of endothelial performance for successful transplantation. Patients with endothelial disorders, who are more exposed to the risk of failure, should be monitored in terms of exposure to other risk factors for graft failure, ensuring the implementation of measures to prevent exposure to modifiable risk factors that may intensify the risk of failure.

The implementation of multidisciplinary institutional protocols that guide the management of patients who will undergo corneal transplantation until they are discharged post-transplantation is an instrument of fundamental importance for the prevention of CGF.

## Supporting information

S1 Table Matching of confounding variables for the patients from the case and control groups. (PDF)

S2 Table Clinical profile of the patients who underwent keratoplasty and corneal graft failure (case and control groups). (PDF)

S3 Table Adequacy of the final logistic regression model. (PDF)
