# Supplementary material for: The donation-transplantation process and corneal graft failure: A case-control study
Source: PLoS One. 2025 May 22;20(5):e0321225. doi: 10.1371/journal.pone.0321225 (PMC12097642; doi:10.1371/journal.pone.0321225)
Supplement: S1 Table — (PDF) [file pone.0321225.s001.pdf]

S1 Table shows the matching effect for each confounding variable in relation to the case and control groups (p=1.00). When looking at the p-value as being equal to 1, one can conclude that the matching was carried out in such a way that the effects of the variables were canceled out.

S1 Table. Matching of confounding variables for patients in the case and control groups. Natal/RN, 2020 (n=81).

**Characteristic**	**Group**		***p***	**Odds ratio [95% CI]**
	**Case n (%)**	**Control n (%)**		
**Vascularization**				
Yes	12 (44.44)	24 (44.44)	**1.000 (1)**	**1.00 [0.39; 2.53]**
No	15 (55.56)	30 (55.56)		
**Glaucoma**				
Yes	06 (22.22)	12 (22.22)	**1.000 (1)**	**1.00 [0.33; 3.04]**
No	21 (77.78)	42 (77.78)		
**Rejection**				
Yes	02 (7.41)	04 (7.41)	**1.000(1)**	**1.00 [0.17; 5.84]**
No	25 (92.59)	50 (92.59)		

Caption: (1) Chi-square test. CI: Confidence interval.
