# Supplementary material for: The donation-transplantation process and corneal graft failure: A case-control study
Source: PLoS One. 2025 May 22;20(5):e0321225. doi: 10.1371/journal.pone.0321225 (PMC12097642; doi:10.1371/journal.pone.0321225)
Supplement: S2 Table — (PDF) [file pone.0321225.s002.pdf]

The clinical profile of the patients is introduced in **S2 Table** below. The variables used in the matching process (vascularization, glaucoma and rejection), although described, had no effect on the inferential analysis due to the control to avoid confounding bias. Accordingly, these variables were not used in the inferential analysis, as they are risk factors for graft failure, as described in the pertinent literature.

S2 Table. Clinical profile of patients who underwent keratoplasty and corneal graft failure (case and control groups). Natal/RN, 2020 (n=81).

Characteristics	n	%
**Type of failure***		
Late	23	85.19
Primary	04	14.81
**Vascularization**		
Yes	36	44.44
No	45	55.56
**Level of vascularization**		
Minimum	15	41.67
Moderate	18	50.00
Intense	03	8.33
**Glaucoma**		
Yes	18	22.22
No	63	77.78
**Rejection**		
Yes	06	7.41
No	75	92.59
**Eye diagnosis**		
Keratoconus	17	20.99
Bullous keratopathy	25	30.86
Fuchs dystrophy	04	4.94
Interstitial keratitis	16	19.75
Leukoma and congenital opacities	10	12.35
Perforation	09	11.11
**Type of disorder**		
Stromal disorders	43	53.09
Endothelial disorders	38	46.91
**Operated eye**		
Right	41	50.62
Left	40	49.38
**Purpose**		
Optical	61	75.31
Tectonic	20	24.69
**Type of keratoplasty**		
Penetrating	71	87.65
Lamellar	10	12.35
**Type of surgery**		
Elective	60	74.07

Characteristics	n	%
Emergency	21	25.93
**Crystalline**		
Phakic	54	66.67
Pseudophakic	25	30.86
Aphakic	02	2.47
**Combined surgery**		
Yes	04	4.94
No	77	95.06
**Previous surgery**		
Yes	38	46.91
No	43	53.09
**Intraoperative complication**		
Yes	09	11.11
No	72	88.89

Caption: [(1)]Cases (n=27).
