# Supplementary material for: The donation-transplantation process and corneal graft failure: A case-control study
Source: PLoS One. 2025 May 22;20(5):e0321225. doi: 10.1371/journal.pone.0321225 (PMC12097642; doi:10.1371/journal.pone.0321225)
Supplement: S3 Table — (PDF) [file pone.0321225.s003.pdf]

S3 Table shows the final multiple logistic regression model using the Backward Wald Stepwise method, which included the following variables: type of disorder and time between enucleation and preservation.

S3 Table. Adequacy of the final logistic regression model. Natal/RN, 2020 (n=81).

**Variables**	***B***	**E.S.**	**Wald statistical test**	**D.F.**	**p-value**	**Exp(*β*)**	**95% CI EXP(*β*)**	
							**L.L.**	**U.L.**
Type of disorder (stromal)	-1.030	0.522	3.893	1	0.048	0.357	0.128	0.993
Time between enucleation-preservation (in minutes)	0.010	0.004	5.738	1	0.017	1.010	1.002	1.017
Constant	0.507	0.672	0.569	1	0.451	1.661		

Note: Stepwise method.
